# Individual identification via electrocardiogram analysis

**DOI:** 10.1186/s12938-015-0072-y

**Published:** 2015-08-14

**Authors:** Antonio Fratini, Mario Sansone, Paolo Bifulco, Mario Cesarelli

**Affiliations:** School of Life and Health Sciences, Aston University, Aston Triangle, B4 7ET Birmingham, UK; Department of Electronic Engineering and Information Technologies, University “Federico II” of Naples, Via Claudio, 21, 80125 Naples, Italy

## Abstract

**Background:**

During last decade the use of ECG recordings in biometric recognition studies has increased. ECG characteristics made it suitable for subject identification: it is unique, present in all living individuals, and hard to forge. However, in spite of the great number of approaches found in literature, no agreement exists on the most appropriate methodology. This study aimed at providing a survey of the techniques used so far in ECG-based human identification. Specifically, a pattern recognition perspective is here proposed providing a unifying framework to appreciate previous studies and, hopefully, guide future research.

**Methods:**

We searched for papers on the subject from the earliest available date using relevant electronic databases (Medline, IEEEXplore, Scopus, and Web of Knowledge). The following terms were used in different combinations: electrocardiogram, ECG, human identification, biometric, authentication and individual variability. The electronic sources were last searched on 1st March 2015. In our selection we included published research on peer-reviewed journals, books chapters and conferences proceedings. The search was performed for English language documents.

**Results:**

100 pertinent papers were found. Number of subjects involved in the journal studies ranges from 10 to 502, age from 16 to 86, male and female subjects are generally present. Number of analysed leads varies as well as the recording conditions. Identification performance differs widely as well as verification rate. Many studies refer to publicly available databases (Physionet ECG databases repository) while others rely on proprietary recordings making difficult them to compare. As a measure of overall accuracy we computed a weighted average of the identification rate and equal error rate in authentication scenarios. Identification rate resulted equal to 94.95 % while the equal error rate equal to 0.92 %.

**Conclusions:**

Biometric recognition is a mature field of research. Nevertheless, the use of physiological signals features, such as the ECG traits, needs further improvements. ECG features have the potential to be used in daily activities such as access control and patient handling as well as in wearable electronics applications. However, some barriers still limit its growth. Further analysis should be addressed on the use of single lead recordings and the study of features which are not dependent on the recording sites (e.g. fingers, hand palms). Moreover, it is expected that new techniques will be developed using fiducials and non-fiducial based features in order to catch the best of both approaches. ECG recognition in pathological subjects is also worth of additional investigations.

## Background

Biometric recognition, often referred as Biometrics, is the science that uses statistical methods to uniquely identify humans by means of their physiological and behavioural characteristics. It is mostly used to solve problems of access control, providing reliable and secure alternatives to the conventional authentication methods [1–5].

Subject identification can be achieved using several human discriminants such as retinal structure, fingerprint, face, palm print, etc. However, each one of them exhibits issues related to the specific hardware to use, the practicability of the measures and the robustness against spoofing attacks. Retinal scan is a relatively quick and secure procedure (there are relatively few chances to forge it), however the technology is still expensive and more importantly the procedure is sometimes perceived as invasive and unpleasant. Fingerprint is the most widespread biometric, it has been used in forensic for about 100 years. Automated systems base their accuracy on a multispectral approach, however simple rolled fingerprint (inked impression images) can relatively easy be forged. The possibility to steal data directly from subjects and the relative ease to replicate them (e.g. silicone fingerprints, pictures or facial masks) posed serious challenges to researchers [6–10] and multimodal recognition systems have been suggested [2, 11–14].

In the last decade, the registration of the electrical activity of the heart on the body surface, namely the electrocardiogram (ECG), has been documented to be suitable for identity recognition [1, 13, 15]. Dedicated research on the ECG analysis has demonstrated its advantages in biometrics: ECG is present in all living individuals, exhibits the typical characteristics of a biometric and it is hard to forge. In addition, ECG analysis is a robust method to detect the aliveness of the subject in authentication scenarios.

To date, many different approaches to human recognition via ECG have been reported in the scientific literature but no agreement exists on the appropriate methodologies. Moreover, the use of ad-hoc signal databases makes difficult the assessment of all existing techniques [16, 17].

This study attempts to provide a survey of the techniques used so far in ECG-based human identification. Here, we present a perspective on the progresses of the last decade’s research in the field and a discussion on the possible implications for future research.

Previous attempts to summarise ECG-based recognition techniques can be traced back to the work of Nasri [18], Odinaka [17] and Israel and Irvine [19].

Nasri briefly summarized the literature by 2008, Odinaka compared the performance of different algorithms testing them on a single database while Israel and Irvine suggested a sensor-based perspective. More recently, some author investigated combination of ECG-traits with other signals (voice, phonocardiography, Laser Doppler Vibrometry) to enhance the identification rate [20–22].

The aim of this survey is to provide a *pattern recognition* perspective, giving a unifying framework for interpreting previous studies and, hopefully, to guide future works. We concentrated on ‘features’ for ECG-based human recognition as well as classification strategies. In addition, we evaluated a weighted mean accuracy of the found journal studies to assess the overall performance of the ECG biometrics techniques used so far.

This survey is organized as follows: section two reports on the search strategy, inclusion criteria, and overall performance evaluation strategy; section three describes the main principles underlying the use of ECG as biometrics, the most spread ECG-based features and the databases used to test algorithms performance; in section four we address the issues of feature selection and dimensionality reduction. Discussion and conclusions report the overall picture dealing with the open issues on ECG-based biometrics.

It is nevertheless worth to highlight what this survey is not aimed at. We will not review ECG pre-processing, which is an established research area and a large number of studies report on efficient methods for noise removal, power-line suppression, baseline-wandering removal etc. [8, 23, 24]. We will also not review the methods for QRS detection, although this is one of the most important issues in all algorithms for individual identification. QRS detection has been reviewed elsewhere and we refer the interested reader to specific papers such as [1, 25–27].

## Search strategy

**Fig. 1:**
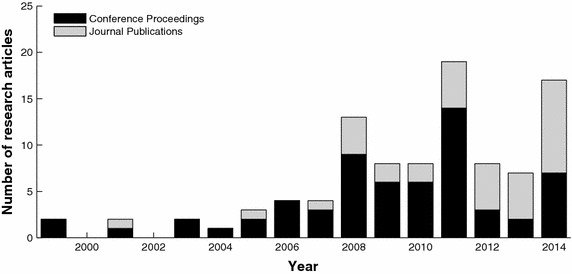
Temporal increase of the research interests on ECG-based biometric recognition.

We searched for papers on the subject from the earliest available date using relevant electronic databases (Medline, IEEEXplore, Scopus, Web of Knowledge). We used the following terms in different combinations: electrocardiogram, ECG, human identification, biometric, authentication and individual variability. The electronic sources were last searched on 1st March 2015. We also performed a hand search of bibliographies of the publications that were found. In our selection we included published research on peer-reviewed journals and conferences proceedings. The search was performed for English language documents. Finally, we retrieved 100 pertinent papers that met the reported criteria. Figure 1 reports the number and the type of research publications during time.

To estimate an overall performance index for ECG biometrics we computed the weighted mean of identification rate by selecting journal publications (18 were considered appropriate due to data availability, use of ECG feature only, etc.). Weighted equal error rate for verification (authentication) scenarios was also computed. The two indexes were obtained by combining the performance of all the studies. Specifically, single study’s performance were weighted (according with the number of subjects involved with respect to the total number of subject of the selected papers) and then added to obtain mean overall performance indexes.

## ECG as a biometric

ECG is the electrical activity of the heart often recorded at the chest level. During its activity, the myocardium—the heart muscle—behaves as a series of connected electric dipoles in a unique fashion called functional “*syncytium*” [28–31]. Heart’s electrical activity is commonly described using an individual time-varying electromagnetic vector [28, 32–36], whose projections can be recorded onto the body surface [32]. Up to twelve specific electrodes positions (leads) are used to monitor heart functions [37, 38], and additional configurations have been proposed for specific purposes [37, 39].

**Fig. 2:**
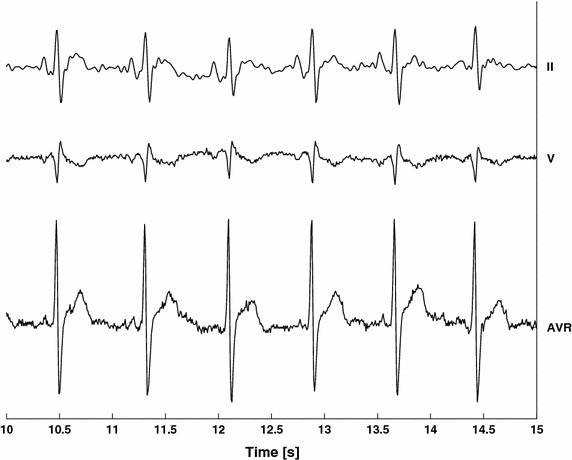
Example of ECG traces from different recording configurations (leads).

Signals recorded from each lead contain different information; however, specific waves, namely P, Q, R, S, T, can be identified within a heartbeat cycle on the different leads (see Fig. 2). Nevertheless, both the time evolution of the dipole vector and its projections onto the subject’s body are influenced by the electrical conduction paths inside the heart, the geometrical characteristics of the heart itself, its position within the chest and also by the inhomogeneity of the conductor volume of the thorax [38].

Therefore, ECG seems to contain enough information for subject recognition. In past studies numerous ‘features’—temporal (locations and intervals among waves), amplitude (height of waves’ peaks) and morphological differences (shapes, proportions, slopes and angles)—have been proposed to recognise individuals.

The hypothesis of the use of ECG as personal identification attribute was suggested by Forsen [40]; however, the first study on the ECG analysis for biometric purposes was carried out by Biel and colleagues [1, 41]: the authors investigated some combinations of features in 12-leads ECG recordings on a sample of 20 subjects.

In the same years moreover, Hoekema and Van Oosterom [42, 43] highlighted and quantified, to some extent, the relevance of the geometrical characteristics in the inter-individual variability of ECG recordings.

Starting from these pioneering studies, ECG biometric literature has grown in the field of pattern recognition (see Fig. 1). Thus, the extraction of appropriate features as well as the classification procedures became both crucial issues.

### Typical realization of an ECG-based identification system

**Fig. 3:**
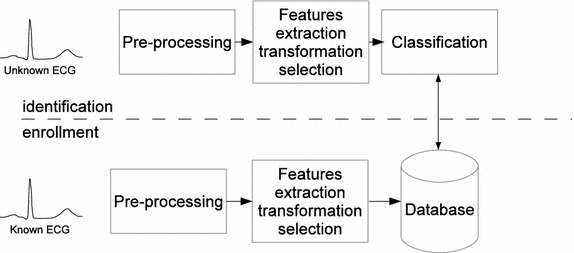
Typical organisation of an ECG-based identification system.

An ECG-based identification system is characterised by a well-defined workflow as depicted in Fig. 3. It firstly requires an *enrolment* phase, which serves to collect and store subject’s distinctive attributes. With the enrolment, specific pre-processing, for noise and artefacts rejection, as well as feature extraction/processing are implemented before the data storage. Once the characteristics of different subjects are stored the *identification* phase can take place. During the identification, in fact, an unknown ECG is presented to the system. As in the enrolment, equal pre-processing and features extraction/transformation are performed. In addition, a specific classification algorithm assigns the extracted features to a *best matching* subject’s data as stored in the database (see “Classifiers”).

Clearly, ECG attributes extraction, selection and transformation, as well as the classifier structure play a fundamental role to achieve the best identification performances. The following paragraphs report the details of each of the mentioned steps.

### ECG features

ECG-based recognition approaches are numerous and very different. ECG attributes (features) are intended to classify the specific subject exploiting inter-subject variability. In general, features are based on the morphology of the heartbeat, on peculiar time intervals derived from ECG waves or on specifically extracted features. The choice of the employed features is generally driven by the complexity of the recognizer, the need of real-time identification, the specific recording device, etc.

No agreement exists on the most appropriate technique or on the type/number of features to consider. Moreover, ECG analysis is often performed on in-house databases making arduous the comparison between techniques.

**Fig. 4:**
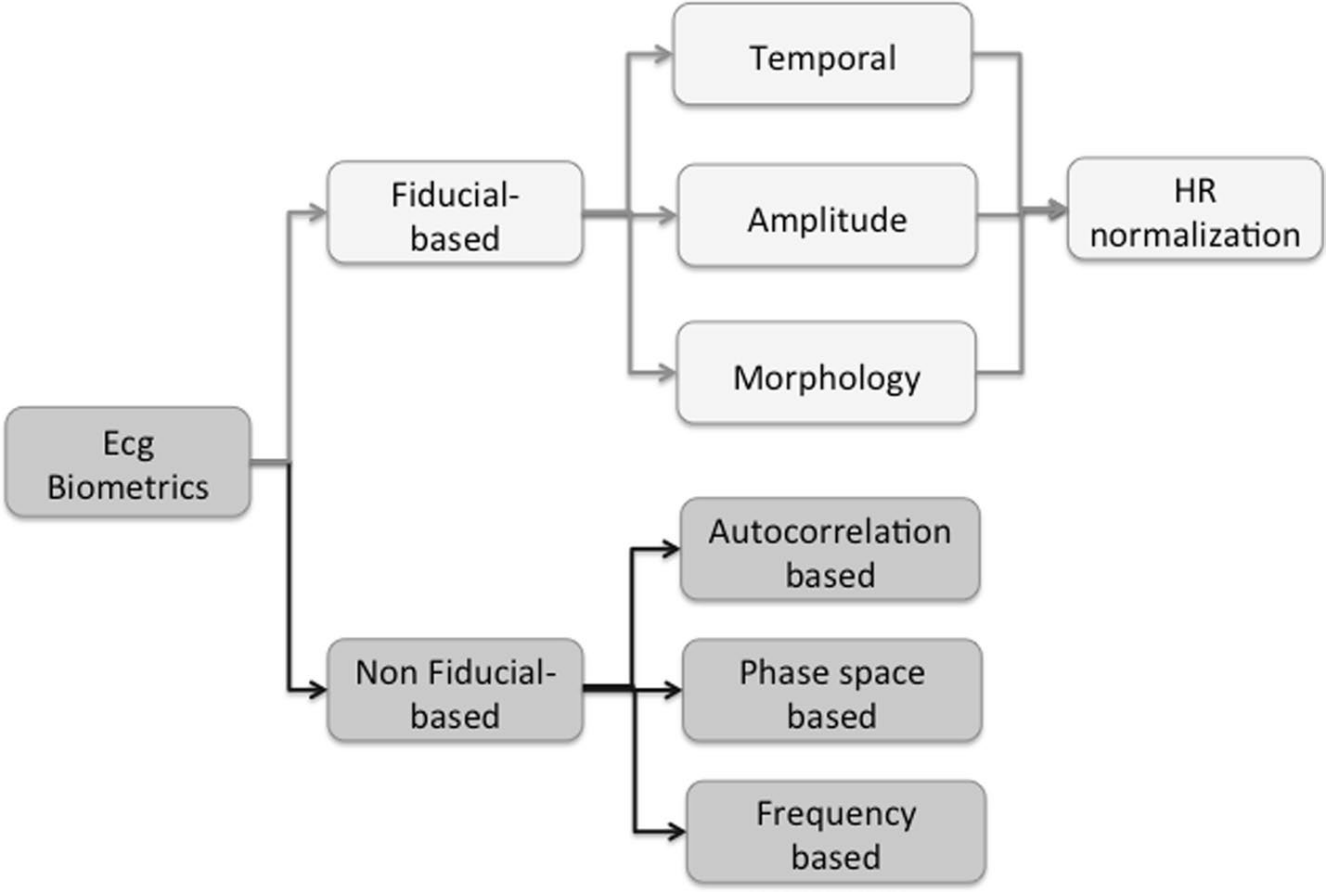
Taxonomy of the ECG-based biometrics analysis.

For the purpose of the present survey, existing approaches have been grouped in two main categories—fiducial based and non-fiducial based—depending on the need to identify precise points in the heartbeat. Each category can be further subdivided as depicted in Fig. 4 by means of the employed features.

In addition, papers in the literature differ as regards the number of the leads used, the recording configuration and the time interval in which the recordings are collected.

#### Fiducial based approaches

By locating specific anchor points on the ECG recordings, namely fiducial points or fiducials, numerous features can be extracted and used as recognizer inputs.

Wave’s peaks, boundaries, slopes or other measures serve as fiducials. Detectors can use adaptive thresholds [44], Fourier synthesis [45], wavelet transform [46, 47], and other approaches as in [48].

Clearly, the extracted features are strictly influenced by the accuracy of the detection. However, in some case, researchers have limited the number of required fiducials (often to the only R peak identification) [28, 31, 35, 47–63].

Fiducials based features can be further subdivided in temporal, amplitude and morphological. Authors generally use these features in combination.

##### Temporal features

The temporal relationships between the various ECG waves (P, QRS, and T) reflect the epochs of heart’s stimulation along its electrical paths starting from the sino-atrial node to the Purkinje fibres and can be used as biometrics discriminants.

**Fig. 5:**
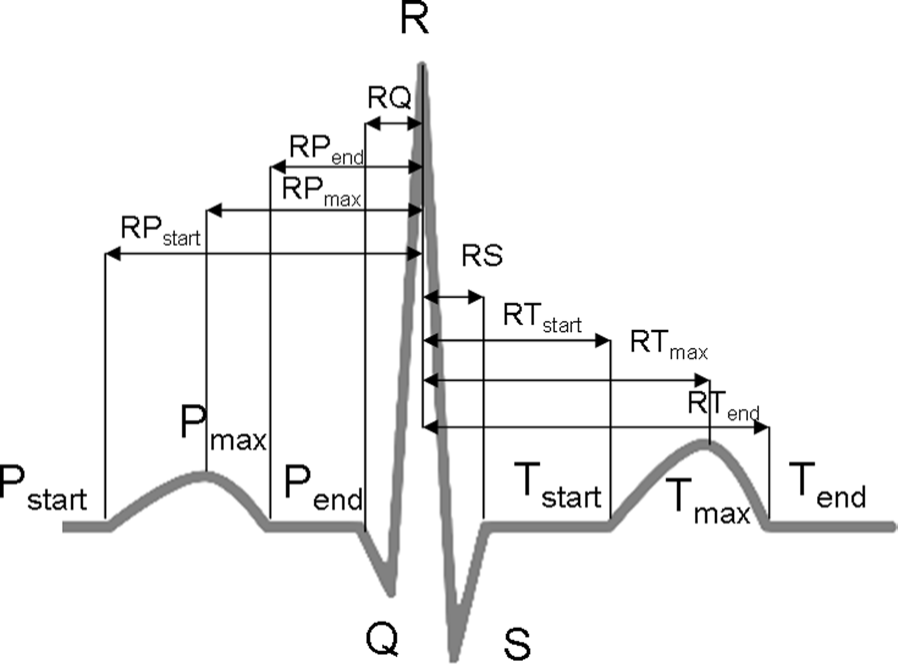
Temporal features: intervals are obtained by locating specific fiducials along the heartbeat signal.

As depicted in Fig. 5, the localisation of specific fiducials allows the computation of several temporal intervals. The most used temporal features include heartbeat wave’s duration (i.e. P, QRS, T) and time intervals between them (PQ, RS, ST, etc.) [1, 2, 4, 5]. RR interval has also been used as fiducial [8].

##### Amplitude features

The inter-individual variability of the amplitude of the heartbeat’s waves can be easily recognised in individuals [2, 13, 14]. Amplitude features capture the relative amplitude between the peaks of an ECG’s wave; they are generally measured relative to the R peak.

**Fig. 6:**
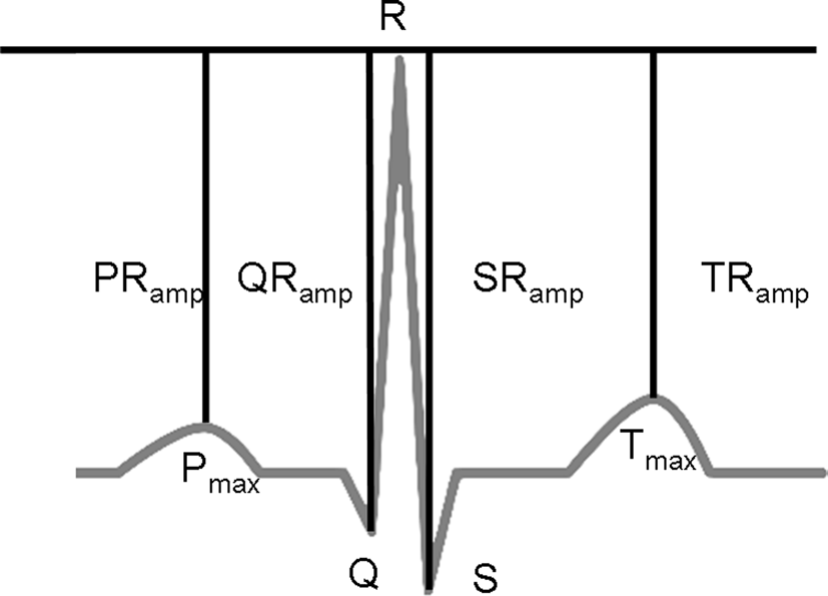
Amplitude features: relative amplitudes can be obtained with respect to the R peak

Amplitude features also include the relative ST segment amplitude [1], the amplitude of peaks of 1st or 2nd derivatives of heartbeat [16] and ratios between them [26]. Figure 6 shows an example of some amplitude features and their location along the ECG trace.

##### Morphological features

Morphological attributes are those carrying information on the shape of the ECG, either as a whole or as its constituent intervals (P-QRS-T). The simplest way to extract morphological features from a heartbeat is to perform the average of the sampled values of specific intervals (e.g. QRS) with respect to multiple aligned (i.e. centred on R peak) heartbeats [47, 48, 51, 52, 55, 57, 59, 64–67]. In this case, the morphological attributes can be considered as a simple extended set of amplitude features.

However, the study of the ECG’s morphology suggested various features: researchers have primarily used slopes among waves, such as ST and RS segment slopes, and angles described by Q, R and S waves [2, 5].

Past studies also concentrate on the specific shape of the whole QRS complex. Palaniappan and Krishnan [8] introduced a peculiar attribute computed from the morphology of the QRS complex. The QRS *form factor* (FF) is defined as in Eq. 1:

1 $$ FF = \sqrt {\frac{{{{\text{var} (x'')} \mathord{\left/ {\vphantom {{\text{var} (x'')} {\text{var} (x')}}} \right. \kern-0pt} {\text{var} (x')}}}}{{{{\text{var} (x')} \mathord{\left/ {\vphantom {{\text{var} (x')} {\text{var} (x)}}} \right. \kern-0pt} {\text{var} (x)}}}}} $$

where x is the QRS complex waveform, *x*′ is the first derivative of the QRS and *x*″ is its second derivative. Biel [1], Shen [26] and Zhang [27] evaluated the suitability of the QRS area. Wübbeler [31], Fang [28] and Sing [30] revealed the characteristic trait of the heart vector by means of studying the whole QRS on different leads.

Polynomial expansions have also been used to synthesize the heartbeat morphology. Khalil and Sufi concentrated on the discrimination feasibility of the Legendre polynomials coefficients derived by fitting the QRS complex [35, 36]. Li [68] used the Hermite interpolation coefficients, Tsao [69] the first order interpolation coefficients while Jun Shen extracted the piecewise linear representation (PLR) coefficients of the entire heartbeat [56]. Specific features (up to 98) were derived via the use of a pulsed triangular wave (Pulse Active Ratio) [70].

The morphology of ECG waves was also investigated by means of wavelet decomposition, frequency analysis and correlation coefficients. Saechia and colleagues focussed on the discriminative characteristics of frequency content of P, QRS and T waves [71]. Hou used the only QRS frequency patterns [72] Tashiro the high frequency components (40–300 Hz) of the entire heartbeat [58]. Lately, Odinaka performed a short time Fourier transform to reveal the spectrogram shape over the heartbeat cycle [54].

Wavelet analysis has been introduced by Chan [48, 49]. Wavelet coefficients were also used by Yao and Wan (Biortogonal wavelets) [47, 59], Chiu (Haar wavelets) [50] and Ye (Daubechies wavelets) [60].

Discrete cosine transform coefficients were also proposed for use in ECG biometrics by Plataniotis et al. [73]. and used by Fattah et al. [74] and Hou et al. [72].

##### Heart rate: based normalization

All the above-described features exhibit a main drawback: the whole heartbeat wave changes with heart rate (HR). This, in turn, induces fluctuations in temporal, amplitude and morphological relationships among waves during physical activity, drug assumption or strong emotions.

Many authors therefore attempted to normalize features with respect to HR changes.

Israel and colleagues [14] assumed the existence of a linear relationship between the heart rate and the features (temporal) computed with respect to the R peak. To remove the dependence on the heart rate, they use a simple normalisation of the features to the length of the entire P-QRS-T complex. Similar to Israel, Kim and colleagues [45] resized the entire heartbeat to a predetermined number of samples n using the inverse Fourier transform.

The approaches adopted by Israel and Kim are however partial. HR influences the timing of heart pulse but it does not homogeneously affect ECG’s waves.

Shen [5, 26] and Singh [30, 75–77] utilized Bazett’s formula to normalize QT interval, while Tawfik [66, 78] and Sansone [79] applied the regression based approach proposed by Sagie [80].

Fatemian and Hatzinakos addressed the heart rate ECG normalization by resampling the T wave to fit a duration of 120 ms (the typical time extension of the T-wave under rest conditions [38, 81]). After T-wave resampling they combined the obtained segment with P and QRS waves in order to compose the whole heartbeat. Finally, the entire heartbeat is resampled to fit 850 ms [64].

#### Non-fiducial based approaches

Temporal, amplitude and morphological features require accurate detection of fiducials and the achieved results are clearly dependent on the recognition procedure. To overcome the problem, new approaches, that do not require fiducials recognition, have been reported.

All the described techniques are based on the assumption that the ECG is a highly repetitive (quasi-periodic) signal. Scientific literature analysis revealed various approaches that we subdivide in three main categories: autocorrelation based, phase space based, and frequency based analyses.

##### Autocorrelation based features

The first attempt to non-fiducial approaches has been proposed by Plataniotis and Hatzinakos [73]. In order to extract valuable attributes, they randomly select 5 s from an ECG recording and estimate the normalized autocorrelation (AC) over a window of *m* lags (see Eq. 2). AC embeds information about ECG’s peculiar characteristics: it is shift invariant and highlights non-random patterns [82]. The QRS complex, in particular, maintains a strong invariance in shape and time width. With this approach, samples that would have to be influenced by fiducials detection, are combined into a sequence of sums of products as given in Eq. 2 reported below:

2 $$ r[m] = \frac{1}{r[0]}\sum\nolimits_{i} {s[i]s[i + m]} $$

In the equation, *r*[*m*] is the AC, *s*[*i*] is the signal at time *i* and *m* is chosen greater than the mean QRS duration (in samples).

Following this first study, Agrafioti [83–86], Wang [87, 88] and other researchers [81–83] also proposed the use of normalized autocorrelation coefficients.

##### Phase space analysis

As for AC based approach, the ECG signal can be characterised in a two-dimensional or even three-dimensional space by using the time-delay technique. The analysis of the phase-space trajectory in fact, can highlight unexplored peculiarities of cardiac activity.

Fang [34] extended the set of features by looking at the three-dimensional vectors of single-lead, time-delayed (4–36 ms), amplitude normalised ECGs $(s(t), \, s(t + dt), \, s(t + 2dt))$.

Then, partitioning the phase-space to a 30 × 30 × 30 grid, he reduced the multi loop trajectory to a coarse-grained features space lowering the computational effort and the loop variability due to noise or ECGs’ irregularity.

Chen [33], and then Coutinho [51] analysed the ECG by characterising its chaotic behaviour. In detail, Chen and colleagues converted three seconds ECG trace to a phase space-plane by using a time delay equal to 20 samples. Then, they computed concise indicators like correlation dimension and Lyapunov exponents as well as the root mean square of the ECG amplitude.

Conversely, Coutinho analyses the recordings similarities using string matching and parsing algorithms. Each recording is converted by applying an 8-bit uniform quantisation, which produces a sequence of symbols (strings) from an alphabet with 2^8^ symbols. Then, a Ziv-Merhav cross-parsing (ZMCP) algorithm [89] is used to evaluate the cross-complexity between different strings. The key idea behind the use of ZMCP is that the cross-complexity becomes lower when the two sequences are similar. Chen et al. explored the use of Lyapunov exponents and correlation dimension [90].

##### Frequency based features

Other authors concentrate on the analysis of ECG’s frequency characteristics [91–95].

Loong et al. [93] utilized a linear predictive technique (linear predictive coding or LPC) to model the frequency content of the ECG recordings. In detail, the model of the spectrum for each subject is obtained considering the first forty points of the linear reconstruction of the ECG spectrum using Eq. 3:

3 $$ \hat{x}[n] = - \sum\limits_{i = 1}^{p} {a_{i} x[n - i]} $$

where the *a*_*i*_ coefficients are evaluated by minimizing the error *e*[*n*] (see Eq. 4) using the Levinson–Durbin recursion [96]:

4 $$ e[n] = x[n] - \hat{x}[n] $$

where *x*[*n*] represent the actual value.

Kouchaki [92] and Zhao [94] used procedures similar to the Hilbert–Huang transform to obtain instantaneous ECG frequency data. The recordings set is reduced to a collection of functions (namely intrinsic mode functions or IMF) with an adaptive process called *Empirical Mode Decomposition* (EMD) [97]. Each IMF is different among individuals and it is not fixed as in Wavelet or Hermitian expansion. IMFs represent simple oscillatory modes of the system under investigation, which can be characterized by means of the Hilbert spectral analysis.

Kouchaki [92] observed that the slowest component of the EMD carries the most of the discriminative information in comparison with other IMF’s.

Zhao and colleagues [94] proposed a modified version of the EMD by using averaged versions of IMFs to raise robustness with respect to noise sources [97]. A consistent number of decompositions is collected for the same ECG by randomly adding white noise to the original signal trait. The corresponding IMFs are then averaged to become noise independent. Finally the spectrum of each IMF is evaluated by Welch analysis. The technique is named *Ensemble Empirical Mode Decomposition.*

Aghakabi [91] and Zokaee [95] use the Mel-Frequency Cepstrum Coefficients (MFCCs) [98]. More in detail, they first select a frame of N samples by using a Hamming window. Then, the Fast Fourier Transform converts the frame from the time domain into frequency domain. A triangular band pass filter bank is applied in the frequency domain to reveal the mel-frequency components [99].

### Multi-lead vs single-lead features

Almost all studies reported in this review addressed the problem of subject identification via ECG using single ECG’s leads. This is mainly due to the usability of ECG-based identification systems. However, since in pattern recognition problems a larger amount of information could raise the probability of a successful recognition [100], multi-lead systems have also been studied.

Biel investigated amplitude and temporal features in 12-leads ECG recordings [1, 41]. He concluded that a single lead is sufficient in assuring good recognition performances, favouring the practical application of the technique (at least three electrodes are needed).

Following the study of Biel, Zhang and colleagues compared the results obtainable with the use of different leads as a single recording [27]. Based on their outcomes they also concluded that the use of few leads is sufficient and in detail, lead V1 and lead V2 can give the best accuracy. These leads grasp a larger ECG signal with respect to other leads since electrodes are placed closer to the heart site.

Moreover, Agrafioti [83, 84] explored the use of the integration of feature extracted from all of the 12 standard measurement leads. However, he found that information integration raises the identification performance only when combining the outcomes of different classifiers at the decision level. Fang et al. [34] used three leads in his phase space trajectory analysis while obtaining comparable results also with a single lead only. Recently Raj and Hatzinakos [101] studied the feasibility of a specific single-arm single-lead on 23 subjects with discrete results (EER 4–12 %).

### Databases

The wide study of ECG signal for clinical purposes favoured the research on the feasibility of ECG as biometric. ECG databases (DBs) have been utilized in the analysis of features and classification performances either public or private (see Table 1). The most used DBs for ECG biometric algorithms testing are available at the Physionet repository [102]. Many researchers used normal and pathological signals DBs: in detail, MIT/BIH’s Normal Sinus Rhythm Database [102], MIT/BIH’s Arrhytmia Database [103], MIT-BIH Supraventricular Arrhythmia Database [104], QT Database [105], Long Term ST Database [106], European ST-T Database [107], Paroxysmal Atrial Fibrillation Challenge Database [108], PTB Database [109]. Conversely, other papers concentrated on private DBs built by recording ECGs with specific devices [1, 14, 16, 28, 35, 45, 47, 48, 54, 59, 65, 86, 93, 110–116].

**Table 1:** Features of the databases used in past studies

References	NS	NL	NL_U_	SF (Hz)	SF_U_	NB	DU (s)	DB
Agrafioti and Hatzinakos [85]	14	12 + 3	1	1,000	1,000	16	na	PTB
Agrafioti and Hatzinakos [83]	13	1 1 12 + 3	1	128 360 1,000	360	16	10	MIT-BIH-NSR MIT-BIH-ARR PTB
	30							
	13							
Biel et al. [1]	20	12	12	na	na	na	na	na
Boumbarov et al. [120]	9	1	1	128	128	12	20	na
Chan et al. [48]	50	1	1	1,000	1,000	12	90	na
Chan et al. [49]	60	1	1	1,000	1,000	12	na	na
Chen et al. [90]	19	1	1	na	na	na	60	na
Fang and Chan [28]	100	2	2	250	250	na	30	na
Fatemian and and Hatzinakos [64]	13	1	1	128 1,000	128 1,000	16	na	MIT-BIH PTB
	14							
Israel et al. [14]	29	1	1	1,000	1,000	na	120	na
Khalil and Sufi [35]	10	1	1	na	na	na	na	na
Kim et al. [45]	10	1	1	200	200	na	30	na
Kyoso and Uchiyama [4]	9	1	1	500	500	12	200	na
Loong et al. [93]	15	1	1	256	256	na	65	na
Lourenco et al. [65]	16	1	1	1,000	1,000	na	120	na
Odinaka et al. [54]	269	1	1	10,000	1,000	na	300	na
Pathoumvanh et al. [132]	10	1	1	500	500	12	200	na
Pereira et al. [112]	77	1	1	256	na	na	600	na
Safie et al. [70]	112	1	1	1,000	1,000	na	30	PTB
Shen and Tompkins [26]	168	1	1	500	500	na	90	MIT-BIH-ARR
Silva et al. [113]	1	1	1	na	na	na	600	na
Singh [77]	50	na	na	na	na	na	na	E-ST MIT-BIH MIT-BIH-ARR MIT-BIH-NSR QT
Singh and Gupta [76]	73	na	na	na	na	na	na	E-ST MIT-BIH MIT-BIH-ARR MIT-BIH-NSR QT
Sriram et al. [114]	17	1	1	na	na	na	720	na
Tawfik and Kamal [78]	22	1	1	500	500	na	10	na
Wan and Yao [59]	38	1	1	na	na	na	240	na
Wang et al. [87]	13	12 + 3	1	1,000 128	na	16	na	PTB MIT-BIH
	13							
Wubbeler et al. [31]	74	3	2	500	na	16	10	PTB
Yao and Wan [47]	20	1	1	na	na	na	na	na
Zhang and Wei [27]	502	4	1	500	na	na	10	na
Zhao et al. [94]	28	na	na	na	250	na	na	MIT-BIH-ST Lomg-term-ST PTB
	86							
	294							

*NS* number of subjects, *NL* number of leads (NL_U_ actually used), *SF* sampling frequency (SF_U_ actually used), *NB* number of bits per sample, *DU* duration, *DB* database used, *na* indicates that information is not available or computable.

## Feature selection

As described in the previous sections, the number of features extracted from the ECG analysis can be very large [1, 28, 34, 52, 60, 74, 93, 112, 117]. However, the information obtained from an extensive set of features is generally redundant. Real-time applications moreover, require a limited number of features in order to allow faster classification. Feature selection is proven to reduce the building and testing time of a classifier by 50 % [118].

The selection of appropriate feature subsets is a critical step in pattern recognition problems; although not all the authors provide feature space dimensionality reduction for ECG based features.

In addition, extracted features are often transformed into new sets by means of linear/nonlinear operators.

Reducing the number of features means identifying the most representative attributes to describe the underlying system/phenomenon.

Israel [14] and Wang [88] used a stepwise canonical correlation [119]. The algorithm starts from one feature adding a new one per iteration; the significance of the features is evaluated by means of Wilks’ Lambda distribution.

Other authors used Principal Component Analysis (PCA) [27, 47, 60, 72, 110, 120] or Linear Discriminant Analysis (LDA) [5, 83, 84, 86, 110, 120, 121] as a feature selection procedure.

PCA reduces the feature space dimensionality by performing Eigen-analysis on the covariance matrix of the original features. The covariance matrix S of a set of data x can be computed as reported in Eq. 5:

5 $$ S = \frac{1}{N}\sum\limits_{i = 1}^{C} {\sum\limits_{j = 1}^{{C_{i} }} {(x_{ij} - \bar{x})} } (x_{ij} - \bar{x})^{T} $$

where *N* is the number of samples, *C* is the number of classes, *C*_*i*_ is the number of samples in the corresponding class, and $\overline{x} = \frac{1}{N}\sum\nolimits_{i = 1}^{C} {\sum\nolimits_{j = 1}^{{C_{i} }} {x_{ij} } }$ is the average of the ensemble. The eigenvectors and associated eigenvalues can be then calculated.

Sorting the associated eigenvalues from the highest to the lowest gives the components in order of significance. Thus, ignoring the components with less significance reduces the feature space dimension.

LDA is a different approach to decrease the dimensionality of a feature set. Given a set of labelled (see “Classifiers”) samples ***x***_*1*_*,…,****x***_*N*_, where ***x***_*j*_ = [*x*_*j1*_*,…,x*_*jp*_] *j* = *1,…,N* is a vector of *p* features, the aim of LDA is to project them on a subspace of *M* < *p* dimensions producing the best possible separation between classes maximizing the *Fisher’s ratio*. In the case of *K* classes, maximisation of this ratio is equivalent to solving the problem of finding eigenvectors and eigenvalues of the matrix ***S***_*W*_^−*1*^***S***_*B*_ and taking the first *M* larger eigenvectors as the directions of the subspace. As ***S***_*W*_ is the within-class covariance matrix, and ***S***_*B*_ is the between-class covariance matrix, this criterion is roughly equivalent to searching for the direction from which the classes have well-separated means and small intra-class covariance. It should be emphasised that LDA must be operated on labelled data (i.e. the class of each element must be known in advance) while PCA can be used when labels are not known. Li [68] introduced a generalized LDA (HLDA) to handle heteroscedasticity of classes in mixed ECG and accelerometer data analysis.

Feature reduction has also been obtained using the information gain ratio analysis (IGR) [122–124]. IGR has been utilized in decision trees learning algorithms to select amongst feature while growing the tree [125]. IGR is based on the concept of entropy: if the feature can assume *c* different values, then the entropy of S relative to this *c*-*wise* classification is defined as in Eq. 6: where *p*_*i*_ is the proportion of S belonging to class *i*.

6 $$ Entropy(S) = \sum\limits_{i = 1}^{C} { - p_{i} \log_{2} p_{i} } $$

Thus, IGR can be defined as the expected reduction in entropy caused by partitioning the examples according to this attribute. The formal definition of the information gain of a feature A, relative to a collection of examples S, is defined in Eq. 7.

7 $$ IG(S,A) = Entropy(s) - \sum\limits_{v \in Values(A)}^{{}} {\frac{{\left| {S_{v} } \right|}}{\left| S \right|}} Entropy(S_{v} ) $$

*Values (A)* is the set of all possible values for attribute *A*, and *S*_*v*_ is the subset of S for which attribute A has value v (i.e., S_v_ = [s ε S|A(s) = v]). Thus, the features are ranked according to their IGR. The selection algorithm begins with an empty set F of best features and then proceeds to add features from the ranked set of features until the classification accuracy begins to drop or it reaches a specific selected value.

Discrete Fourier Transform (DFT), discrete wavelet transform (DWT) or discrete cosine transform (DCT) have also been used to provide concise set of coefficients. With these approaches, the selected features are the coefficients of the transformation that result significantly different from zero [50, 73, 83, 84, 87].

## Classifiers

In pattern recognition problems, classification strategies are the ways with which a vector of analysed features is assigned to a specific subject. Given a vector **x** = [x_1_,x_2_,…,x_p_] composed by features extracted from the ECG of an unknown subject, the aim of the classifier is to assign **x** to the correct subject. To this aim, a data-set of samples should be available. The data-set is constituted of labelled feature vectors (x_j_, L) where L is the class (subject) label of the sample j = 1,…,N. Typically, a large number of samples should be available per each subject. In order to evaluate the performance of the classifier (error rate) typically the data-set is divided into a training-set and a test-set. The training-set is used for classifier design while the test-set is used for performance assessment (see “Discussion”).

### Distance based classification

Classifiers can be designed on the basis of several approaches. One common approach leads to assign the unknown sample to the class of the closest sample in the features space. [5, 28, 31, 35, 45, 48–50, 53, 65, 66, 71, 73, 75, 78, 83–85, 87, 88, 95, 111, 112, 114, 122–124, 126–129].

The distance between two feature vectors **x**_1_, **x**_2_ is typically measured using Euclidean norm d(**x**_1_, **x**_2_) = ||**x**_1_ − **x**_2_||^2^. A variant of this approach involves preliminary computation of a *template* (or prototype) **μ**_*j*_ per each class *j* (typically a samples average): the unknown sample is assigned to the class of the closest template (*template matching*).

However, Euclidean norm does not account for sample distributions with unequal variances in different directions. In that case, assuming that all classes share a single covariance matrix **Σ**, a better approach could be to use the minimum Mahalanobis’ distance $d\left( {x,\mu_{i} } \right) = \left( {x - \mu_{i} } \right)^{T} \varSigma^{ - 1} \left( {x - \mu_{i} } \right)$ between the unknown vector **x** and **μ**_***i***_ is the mean (*template*) of the *ith* class [16].

Another common approach is to construct N ‘discriminant functions’ g_i_(**x**), i = 1…N, one per each class: the unknown vector is assigned to class *k* if g_k_(**x**) > g_i_(**x**) i = 1…N. The discriminant functions can be constructed, for example, using a Bayesian approach. As known, the Bayes formula relates the a posteriori probability that to the a priori distribution of classes and to the *likelihood* of the features, given a specified class *k* (see Eq. 8)

8 $$ p\left( {\omega_{k} |x} \right) = \frac{{p(x|\omega_{k} )p(\omega_{k} )}}{p(x)}. $$

Using this approach with a Gaussian distribution of features, we obtain the discriminant function [27] reported in (9):

9 $$ g_{i} (x) = - \frac{1}{2}\left( {x - \mu_{i} } \right)^{T} \sum\limits_{i}^{ - 1} {\left( {x - \mu {}_{i}} \right) - \frac{1}{2}\ln \left| {\varSigma_{i} } \right|} $$

where **x** is an unknown feature vector, **Σ**_*i*_ is the covariance matrix of the features of *i*th class, **μ**_*i*_ is the template of the *i*th subject.

Another comparison metric is the *maximum correlation* between two signals (vectors) [64], which exhibits the amount of similarity between two signals. The correlation is defined as in Eq. 10

10 $$ \rho_{xy} [m] = \frac{{\sum\nolimits_{i = 0}^{N - \left| m \right| - 1} {x[i]y[i + m]} }}{{\sqrt {E_{x} E_{y} } }} $$

where* x*[*i*] and* y*[*i*] *i* = *1,…,P*, represent two different ECG signal windows of length *N*, $E_{x} = \sum\nolimits_{i = 0}^{N} {x^{2} [i]}$ is the energy of the signal. It achieves the maximum value for m = 0.

Israel [14] and Shen [5] used the LDA technique [130]. LDA was born as a method for dimensionality reduction; however it can also be used for classifier design.

Chan [48] used three different distances: percent residual difference (PRD) that quantifies the amount of differences between two ECG with respect to the variability contained in the unknown ECG; correlation coefficient (CCORR) that measures the least squares fitting of the two ECG to be compared and WDIST that measures the difference between the discrete wavelet coefficients of the two ECGs.

### Neural networks

In the context of subject recognition by ECG processing, Neural Networks (NN) have been used in [8, 33, 58, 59, 66, 69, 71, 78, 90, 93, 120, 124, 131]. The most successful NN is the Multi Layer Perceptron (MLP). In contrast to the conventional approaches seen in the previous section, MLP is capable to solve complex non-linear classification problems.

**Fig. 7:**
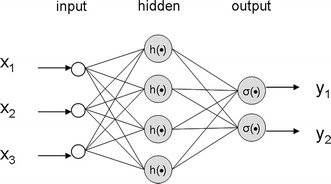
A schematic structure of a multi-layer perceptron (MLP) neural network.

Figure 7 depicts a typical MLP: each node (i.e. neuron) has a non-linear activation function acting upon the (features) inputs **x** **=** [x_*1*_,…,x_*p*_]: in particular, each input has a (synaptic) weight **w**_j_^T^ = [w_*j1*_,…,w_*jp*_], in order that the output z_*j*_ of the neuron *j* is the action of the activation function on a linear combination as z_*j*_ **=** h(**w**_j_^T^**x**). Therefore, the output of the system is given by z_*j*_ **=** σ(**w**_j_^(2)T^ h(**w**_j_^(1)T^**x**)) where the superscript (1) refer to the first (hidden) layer of neurons, and the superscript (2) refer to the second (output) layer of neurons.

While a single layer NN can solve only linearly separable problems (sample are separable via a hyperplane in the features space), a hidden layer is typically added to give the MLP the capability to solve non-linearly separable problems (Fig. 7).

### Non-conventional approaches

Wang et al. [87] used nearest neighbour (NN) classifiers in combination with Euclidean distance. However, they used a hierarchical approach and features integration. More importantly, they introduced a new approach in this field: the hierarchical approach that divides the problem in two sub problems (large class to small class problem): first, they used a first-level classification based on analytic features only (time + amplitude of fiducial points), then used PCA based classification module to classify subjects that can be potentially confused by the first stage!!

Singh and Singh used a different approach [30]. Per each subject *j* in the database they constructed a Pattern Matrix **P**^(j)^ in the following way. Per each subject they ‘arbitrarily’ selected a number *m* of heartbeats. Per each heartbeat they extracted *p* features $x_{k}^{(j)} = \, \left[ {x_{k1}^{(j)} , \ldots ,x_{kp}^{(j)} } \right]$ where *k* = *1,…,m*: the latter are the rows of the Pattern Matrix. Given an unknown vector **x** the distance score measure $s^{{(j)}}  = \frac{1}{m}\sum\nolimits_{{k = 1}}^{m} {s_{k}^{{(j)}} }  = \frac{1}{m}\sum\nolimits_{{k = 1}}^{m} {} \sum\nolimits_{{r = 1}}^{p} {\left| {x_{{kr}}^{{(j)}}  - x_{r} } \right|}$ a smaller score indicates a good match.

## Discussion

The field of biometric recognition via the use of ECG characteristics is certainly engaging and results seem encouraging. According with Table 2 the weighted mean identification rate is equal to 94.95 % and the overall equal error rate (in an authentication scenario) to 0.92 %.

**Table 2:** Estimation of the overall performance of the use of ECG as biometric

References	IR (%)	AEER	IRW	AW	WIR (%)	WA (%)
Agrafioti and Hatzinakos [85]	100.00	na	0.010	na	0.61	na
Agrafioti and Hatzinakos [83]	96.20	0.87	0.020	0.10	2.35	0.08
Biel et al. [1]	98.00	na	0.010	na	0.86	na
Boumbarov et al. [120]	86.11	na	0.000	na	0.34	na
Chan et al. [48]	89.00	na	0.020	na	1.94	na
Chan et al. [49]	100.00	na	0.030	na	2.62	na
Chen et al. [90]	91.20	na	0.010	na	0.76	na
Fang and Chan [28]	95.00	na	0.040	na	4.15	na
Fatemian and Hatzinakos [64]	99.63	na	0.010	na	1.17	na
Israel et al. [14]	100.00	na	0.010	na	1.27	na
Khalil and Sufi [35]	na	na	na	na	na	na
Kim et al. [45]	Na	na	na	na	na	na
Kyoso and Uchiyama [4]	94.20	na	0.000	na	0.37	na
Loong et al. [93]	100.00	na	0.010	na	0.66	na
Lourenco et al. [65]	94.30	10.10	0.010	0.03	0.66	0.28
Odinaka et al. [54]	99.00	0.37	0.012	0.46	11.63	0.17
Pathoumvanh et al. [132]	97.00	na	0.000	na	0.42	na
Pereira et al. [112]	99.00	0.70	0.030	0.13	3.33	0.09
Safie et al. [70]	93.60	na	0.050	na	4.58	na
Shen and Tompkins [26]	95.30	na	0.070	na	6.99	na
Silva et al. [113]	na	na	na	na	na	
Singh [77]	82.00	0.10	0.030	0.13	2.61	0.01
Singh and Gupta [76]	99.00	na	0.020	na	2.16	na
Sriram et al. [114]	97.00	15.00	0.010	0.03	0.72	0.44
Tawfik and Kamal [78]	99.08	na	0.010	na	0.95	na
Wan and Yao [59]	100.00	na	0.020	na	1.66	na
Wang et al. [87]	100.00	na	0.010	na	1.14	na
Wubbeler et al. [31]	99.00	0.03	0.030	0.13	3.20	0.00
Yao and Wan [47]	91.48	na	0.010	na	0.80	na
Zhang and Wei [27]	97.40	na	0.220	na	21.35	na
Zhao et al. [94]	95.00	na	0.180	na	16.93	na
Total					96.22	1.16

*IR* identification rate, *AEER* equal error rate for authentication scenarios, *IRW* IR weights, *AW* AEER weights, *WIR* weighted IR, *WA* weighted AEEE, *na* indicates that information is not not available or not computable.

Our results pointed out that subject identification depends primarily on the choice of the utilised feature(s). Fiducial based approaches benefit of well-established normalization algorithms to compensate for changes in ECG signal due to the heart rate variability [111, 132] but they are commonly affected by the performance of the fiducials detection algorithms.

Non fiducial based approaches, conversely, offer a promising alternative to reduce error rate and computational effort. They do not require the identification of ECG waves and have the advantage to potentially take into account *fine* features which could be lost using fiducials.

The choice of number of leads influences the recognizer outcomes. Many authors investigated the performances of multi lead systems, others have also investigated ECG related signals [20, 22, 133]; however, we believe that the feasibility of biometric identification via ECG analysis could only be obtained by limiting the number of required leads/signals: to this regard, single ECG lead systems would be desirable.

The time span in which the selected features are effective is also an important matter of research. Most of the published studies considered the capacity of a system to identify a subject only at a specific time; few papers addressed the variability of ECG features with time or with physiological (e.g. subject’s aging, stress, activity, etc.) or pathological conditions [79, 134–140].

Lastly, studies generally rely on post hoc analyses, although some real time examples have been reported [136, 141, 142].

## Conclusions

Four major issues must be highlighted regarding the adequacy of the studies conducted so far. First, while a great effort has been spent in feature selection and classifier design, it is not yet clear what is the best set of features and classification scheme for ECG biometrics (hierarchical, ensemble etc.). Non-fiducial based techniques can reduce the computational effort as well as the error rate due to the ECG waves recognition. Therefore, it is expected that the new techniques to be developed will use fiducials and non-fiducial based features in order to catch the best of both approaches. Further analysis should be addressed on the use of single lead recordings and the study of features which are not dependent on the recording sites (e.g. fingers, hand palms).

Second, as regards the population size, the majority of the studies have been conducted on a small population (about a few tens of subjects). Therefore, the applicability of ECG biometric recognition on a large scale (real life authentication scenario) it is not yet proven.

Third, almost all studies (except for [17] and [31] ) ignored the variability of the ECG during life span (i.e. variability induced by work, ageing, iterate sport activity etc.); moreover, only few studies [57, 83, 136] considered the applicability of these techniques when subjects suffer from pathological conditions. ECG recognition in pathological subjects is another aspect worth of additional investigations.

Fourth, it must be emphasised that, while guidelines are available for ECG acquisition in the clinical scenario, there is still a lack of standardisation on ECG acquisition (number of leads and their positioning, sampling frequency, number of bits, filtering, type of electrodes, number of leads etc.) for biometrics applications. However, ECG databases for biometric recognition should ideally include recordings, at a given sampling frequency and conditions, from the same subjects in different circumstances (e.g. relaxed, during and after physical training) and along a period of several years.

If addressed, the mentioned challenges will contribute to move this promising technique from its state of adolescence to a proper daily life adoption.
